# Task-specific machine learning system for automatic assessment of multi-limb bradykinesia in Parkinson's disease

**DOI:** 10.3389/fnins.2026.1774097

**Published:** 2026-04-10

**Authors:** Yiyuan Zhang, Qiangqiang Chen, Huijuan Wu, Chen Chen, Wei Chen

**Affiliations:** 1 Department of Biomedical Engineering, College of Biomedical Engineering, Fudan University, Shanghai, China; 2 Department of Neurology, Changzheng Hospital, The Naval Medical University of Chinese PLA, Shanghai, China; 3 Center for Medical Research and Innovation, Shanghai Pudong Hospital, Fudan University Pudong Medical Center, Shanghai, China; 4 Human Phenome Institute, Fudan University, Shanghai, China; 5 School of Biomedical Engineering, The University of Sydney, Sydney, NSW, Australia

**Keywords:** bradykinesia, feature engineering, machine learning, Parkinson's disease, signal processing, wearable technology

## Abstract

**Introduction:**

Bradykinesia, a cardinal physical dysfunction of Parkinson's disease (PD), is generally evaluated by Section III of the Movement Disorders Society-sponsored revision of the unified Parkinson's disease rating scale (MDS-UPDRS). The evaluation process requires the supervision of clinicians; therefore, the results may be subjective and increase clinicians' workload.

**Methods:**

To compensate for these drawbacks, this study proposes a task-specific machine learning system that incorporates wearable sensors to automatically monitor bradykinesia. Initially, this study enhanced the peak detection algorithm by considering specific motion characteristics, such as movement amplitude, to make it more adaptable to individualized signals. Furthermore, a task-specific score prediction system was proposed, incorporating the optimal sensor placement positions and appropriate score prediction algorithms. Specifically, seven machine learning models and four ensemble methods were explored for each of the five MDS-UPDRS III tasks.

**Results:**

The system was tested in 21 patients and eight control individuals. The performance of the system was evaluated under three scenarios: precise prediction (0 vs. 1 vs. 2 vs. 3), abnormal/normal [0 vs. (1, 2, 3)], and normal-moderate/severe [(0,1,2) vs. 3], with the highest average F1 score reaching 0.8722 and the lowest total root mean square error at 0.3214 among tasks in critical status prediction. Furthermore, this study, through statistical analysis, suggested specific scoring features tailored to each task.

**Discussion:**

This study demonstrated the feasibility of accurately and automatically monitoring bradykinesia of PD.

## 1 Introduction

Bradykinesia is a hallmark motor dysfunction symptom of Parkinson's disease (PD) (Dauer and Przedborski, 2003). It refers to a wider variety of motor deficits, involving decreased movement amplitude (hypokinesia) and a progressive decrease in velocity and amplitude during repetitive movements (sequence effect) (Goetz et al., 2008; Berardelli, 2001). It typically occurs on one side of the body and then likely on both sides as the disease progresses (Sveinbjornsdottir, 2016; Hoehn and Yahr, 1967). Consequently, it can affect the performance of daily activities, such as eating and writing, ultimately reducing quality of life (Dauer and Przedborski, 2003; Sveinbjornsdottir, 2016). Thus, accurate assessment of bradykinesia is crucial in the diagnosis of PD, the development of treatment plans, and the monitoring of disease progression.

Clinically, bradykinesia is evaluated in Section III of the Movement Disorder Society-sponsored revision of the unified Parkinson's disease rating scale (MDS-UPDRS) (Goetz et al., 2008). Within this scale, five tasks are involved to assess the different bradykinesia extremities, namely finger tapping (FT), hand movements (HM), pronation-supination movements (PS), toe tapping (TT), and leg agility (LA). The results of these individual tasks are then combined to form the global bradykinesia. However, the examination process requires the subjective judgment of experienced clinicians, resulting in low inter-rater reliability due to its inherent subjectivity and a reduced frequency of assessment. In addition, patient performance could be affected by white coat hypertension. Consequently, these drawbacks underscore the need for an efficient and objective motor function assessment tool (Tian et al., 2024; Guo et al., 2020).

Driven by the rapid evolution of sensor technology and machine learning (ML), automatic evaluation systems for bradykinesia have been explored. Multiple types of sensors, such as video (Islam et al., 2023; Guarín et al., 2024; Guo et al., 2020; Morinan et al., 2023; Lin et al., 2025), force plates (Exley et al., 2022), wearables (Patel et al., 2009; Meng et al., 2025; Peng et al., 2025; Cakmak et al., 2022; Sánchez-Fernández et al., 2023), surface electromyography (sEMG) (Lin et al., 2025), etc., have been utilized. Among these sensors, wearables, typically inertial measurement units, pose less privacy concerns, and the collected signals are closely related to the movement of body parts. (Cakmak et al. 2022) proposed monitoring the UPDRS III scores through the three acceleration characteristics of the single PS task (speed, frequency, and acceleration). These characteristics were found to have statistically strong negative correlations with bradykinesia, rigidity, and gait or postural instability. (Sánchez-Fernández et al. 2023) utilized accelerometers (ACMs) comprised with gyroscopes (GYRs), and magnetometers to monitor the movement of PS. With a K-nearest neighbors (KNN)-autoencoder model, they obtained an accuracy of 0.89 in score prediction. However, the single task is limited to representing the state of the whole body, and the occurrence of symptoms is individualized.

(Crowe et al. 2024) developed a convolutional neural network (CNN) to predict global bradykinesia using ACMs and GYRs obtained from the wrist and ankle (accuracy at 75%), but did not predict the severity level. Furthermore, using a deep learning (DL) algorithm (XceptionTime), (Singh et al. 2023) classified the severity of the five UPDRS tasks into low (scores of 0 or 1) and high (scores of 3 or 4). The average accuracy reached 0.812 with the maximum vote inference. These DL algorithms are effective in extracting features directly from raw signals. However, the kinematic meaning of the features needs to be further explored, due to their end-to-end mechanism. Furthermore, since (Singh et al. 2023) did not consider the severity at 2, their results do not apply to the real clinical situation.

To overcome the aforementioned limitations, the present study aims to explore a wearable embedded task-specific system to automatically assess the five UPDRS tasks. The main contributions of this study are:

- Improving the automatic multiscale-based peak detection (AMPD) algorithm (Scholkmann et al., 2012) to uniquely incorporate personalized motion signatures. With the proposal of three novel adaptive filtering mechanisms, we improved the AMPD to dynamically adapt to individual movement characteristics, such as personalized amplitude and velocity information, enabling unprecedented accuracy in detecting motion peaks from varied signals due to the complexity of tasks and PD severity levels.
- Proposing a task-specific ML system to thoroughly and automatically evaluate the performance of five UPDRS III tasks. Firstly, to better explore the pathophysiological mechanisms of bradykinesia of each extremity, especially the extremity on different body sides, four groups of representative characteristics (kinematic, physiological, time domain, and frequency domain) were investigated for each task, and their importance was analyzed. Secondly, the task-specific algorithm was explored among seven ML models combined with four ensemble methods.
- Exploring the appropriate sensor placement position for each task. The performance of different sensor placement positions in monitoring the same task was compared to investigate the most appropriate positions for each task.

This article is managed as follows. Section 2 introduces the dataset, the signal processing component, the feature extraction techniques, and the machine learning algorithms. Section 3 lists the score prediction results. Section 4 discusses the results, analyzes the features, and proposes future research directions, while Section 5 is the conclusion.

## 2 Materials and methods

**Figure 1:**
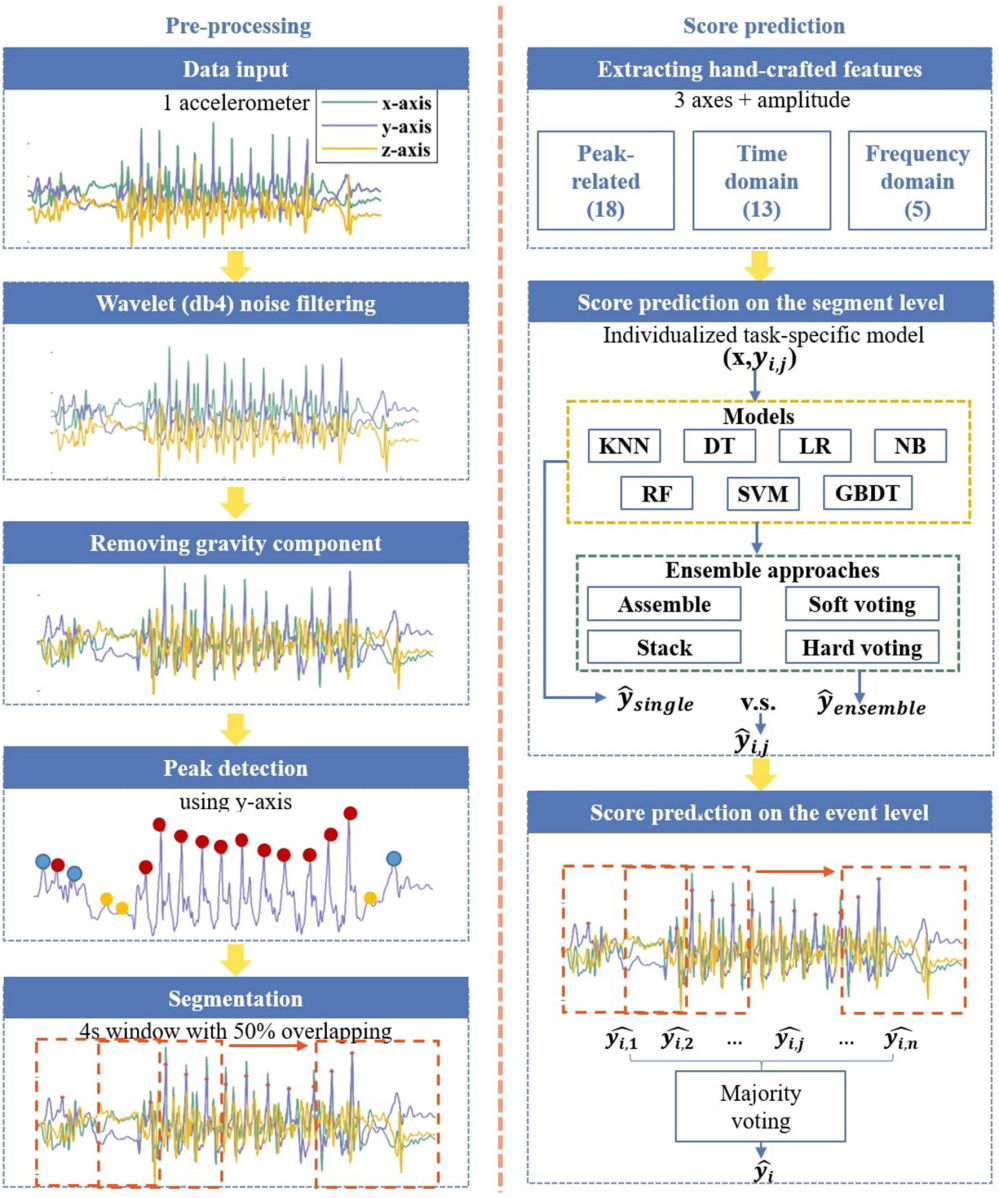
Proposed task-specific score prediction process. In the peak detection step, red spots indicate detected peaks, green spots indicate flat peaks, and yellow spots indicate peaks with lower amplitude.

Figure 1 illustrates the proposed task-specific score prediction process. This process consists of two sections: signal pre-processing and automatic score prediction. Detailed information on the system is introduced below.

### 2.1 Dataset

This study analyzed the public data collected by (Harrigan et al. 2020). Twenty-one patients with PD (male: female = 15:6), aged 66.5 ± 12.34 years, and eight control individuals (male: female = 5:3), aged 63.88 ± 7.45 years, participated in the experiment. Under the researchers' instruction, every participant performed the five UPDRS tasks once. Among them, 10 patients and all control individuals participated in a retest session one week or multiple months later during follow-up, for a total of 47 records for each task. Detailed information is illustrated in Table 1. The task performance was rated on a scale of 0–4, where higher scores indicated greater impairment (0 = normal; 4 = unable to perform the task). Because the number of tests scored as 4 was small (a maximum of four times in the left side TT), these tests were categorized as 3 in the later data analysis. To be noted, only one unique score was provided for each task; thus, it was not possible to discuss inter-rater reliability. Eight triaxial ACMs (sampled at 80 Hz) were attached to each side of four body parts. For analysis, nonetheless, we focused exclusively on the most relevant sensors: index fingers for FT/HM, wrists for PS, big toes for TT, and ankles for LA.

**Table 1:** Number of records for every task at five scores in PD patients and control individuals.

Score		PD patients					Control individuals				
Task		0	1	2	3	4	0	1	2	3	4
FT-R	Test	1	11	3	6	0	5	3	0	0	0
	Retest	4	3	2	1	0	5	2	1	0	0
FT-L	Test	1	8	6	5	1	3	5	0	0	0
	Retest	1	4	3	2	0	2	5	0	1	0
HM-R	Test	4	8	4	5	0	2	4	2	0	0
	Retest	2	3	2	3	0	5	2	0	1	0
HM-L	Test	3	7	5	5	1	2	5	1	0	0
	Retest	2	2	2	3	1	3	4	1	0	0
PS-R	Test	0	10	4	7	0	4	3	1	0	0
	Retest	0	4	4	2	0	5	1	2	0	0
PS-L	Test	0	5	8	7	1	1	4	2	1	0
	Retest	0	1	2	6	1	1	3	4	0	0
TT-R	Test	0	9	4	4	2	2	5	1	0	0
	Retest	1	3	4	2	0	3	4	1	0	0
TT-L	Test	0	7	5	4	3	2	4	2	0	0
	Retest	0	2	3	4	1	2	4	2	0	0
LA-R	Test	3	10	2	3	1	6	1	1	0	0
	Retest	3	5	1	1	0	5	2	1	0	0
LA-L	Test	3	10	2	2	2	5	1	2	0	0
	Retest	0	6	2	2	0	3	5	0	0	0

FT, finger tapping; R, right side; L, left side; HM, hand movement; PS, pronation-supination movements; TT, toe tapping; LA, leg agility.

### 2.2 Signal pre-processing

#### 2.2.1 Forms of signals

**Figure 2:**
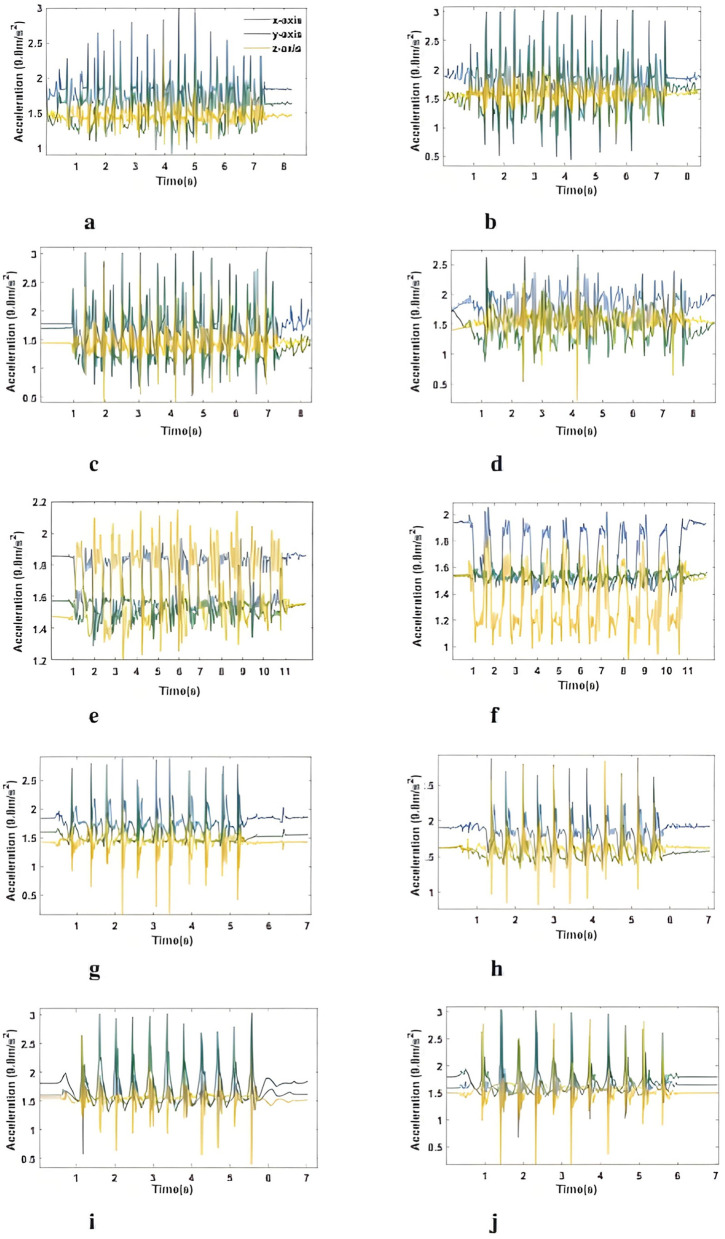
Example of acceleration signals of every task. The signals are of participant 1. **(a)** FT-R. **(b)** FT-L. **(c)** HM-R. **(d)** HM-L. **(e)** PS-R. **(f)** PS-L. **(g)** TT-R. **(h)** TT-L. **(i)** LA-R. **(j)** LA-L.

As illustrated in Figure 2, the movement patterns of all tasks can be categorized into two types. The first type is linear movement (FT, HM, TT, and LA), which is the combination of two high-frequency peaks. These two peaks separately belong to movements; the peak with higher amplitudes represents the start of the index finger tapping the thumb, and the one with lower amplitudes represents the start of opening the fingers, as suggested by (Stamatakis et al. 2013). The second type is typically related to PS, which is the rotational movement in the form of a square wave, as proposed by (Cakmak et al. 2022).

#### 2.2.2 Noise filtering

To remove the noise caused by motion artifacts and maintain the detailed information of the movement (especially the high-frequency peaks), the original acceleration signals were filtered using the Daubechies wavelet transform at level 4 (db4), following the study (Stamatakis et al., 2013). Afterwards, the gravity component was filtered out by subtracting the signal's average value.

#### 2.2.3 Peak detection

In UPDRS, the assessment guideline for these tasks is based on three criteria: a steady rhythm without interruptions or hesitation, slowness, and amplitude decrements (Goetz et al., 2008). To capture these criteria, the initial step was to find peaks, specifically the peak with a higher amplitude in the linear movement and the peaks in the rotational movement. The AMPD algorithm was used to detect peaks by analyzing local maxima on a multiscale (Scholkmann et al., 2012). This algorithm is well-suited for fitting periodic signals, particularly those with changes, such as interruptions or slowdowns, and is robust to noise at multiple frequencies. Moreover, it does not require manually setting any hyper-features. The example of the detected peaks is shown in Figure 1.

In this study, the AMPD algorithm was employed to find peaks in the acceleration of the y-axis, as it was more sensitive to the onset of motion with the most significant variations compared to the other two axes, as shown in Figure 2. However, due to the subjective differences, for example, patients at different severity levels, and the complexity of the tasks, for example, two peaks within one complete movement of finger tapping for opening and closing fingers, respectively, the AMPD algorithm still had limitations in this dataset. Thus, the detected peaks were further evaluated following three rules.

- Evaluation of the first or last peak. As illustrated in Figure 1, although it is a local maximum, the first peak occurs at the first timestamp. Therefore, it is incomplete, and its validity remains uncertain; hence, it is removed.
- Removal of peaks with lower amplitudes. Figure 1 presents the peaks with significantly lower amplitudes compared to their neighboring peaks. These may have been related to motor disturbances, such as tremors or brief pauses and starts during movement. To remove these, a threshold value of 0.3 times the amplitude of the highest peak was set.
- Removal of flat peaks. After removing the peaks with lower amplitudes, there still existed peaks with lower prominence due to unstable movement. To achieve this, the first derivative of the signal was computed, and then its absolute value was taken. Next, the integral within a defined neighborhood of ± two data points was calculated. In the integrated signal, if the amplitude at the original peak location exceeds 0.1 of the highest amplitude, this peak was retained.

#### 2.2.4 Signal segmentation

The filtered signals were segmented with a sliding window. After trials of window lengths of (1, 2, 3, 4) s and overlapping rates of (0.25, 0.5, 0.75), the optimal window length was set to 4 s with a 0.5 overlap.

### 2.3 Feature extraction selection, and analysis

Thirty-eight categories of hand-crafted features were extracted from the segments. As listed in Table 2, features were grouped into four categories: kinematic (18), time domain (13), frequency domain (5), and physiological features (2). The kinematic features were related to the alteration of single movements (intervals), which originated from studies (Arora et al., 2015; Stamatakis et al., 2013). In addition to speed, the other 17 features were extracted from the duration of the intervals and the amplitude of the peaks. The time- and frequency-domain features were extracted from each axis, as well as the amplitude of the signal. Besides the conventional features, such as the mean, standard deviation, and kurtosis, some characteristics were typically proposed to evaluate bradykinesia, namely spectral entropy and flatness (Mahadevan et al., 2020). The physiological factors, including age and gender, were also included as features since they have been verified to be relevant to the severity of bradykinesia (Picillo et al., 2017).

**Table 2:** Information on features.

Kinematic (interval, amplitude of the peaks)		Time-domain (x-, y-, z-axis, magnitude)	
Speed	Mean	Root mean square (RMS)	Range
Median	Standard deviation (STD)	Entropy	Cross correlation
Skewness (SK)	Kurtosis (KU)	Interquartile of auto-covariance (IQR)	Zero crossing rate (ZCR)
Range	Coefficient of variation (CV)	SK	Slope Sign Changes (SSC)
Mean Square Value (MSE)	Autoregressive coefficients (ARC)	Mean	STD
Teager-Kaiser energy operator (TKEO)	Detrended fluctuation analysis (DFA)	KU	Variance (Var)
Fatigue1	Fatigue2	Harmonic mean (H)	
Fatigue5	Number of intervals (NI)	**Frequency domain (x-, y-, z-axis, magnitude)**	
Appearance of frequency ascending (AscdF)	Appearance frequency descending (DscdF)	Main amplitude (MainAmp)	Main frequency (MainF)
**Physiological**		Spectral flatness (SpecF)	Spectral Entropy (SE)
Age	Gender	Mean Frequency (MeanF)	

For each task, the most representative features were selected via grid searching. Initially, features were ranked in descending order based on their importance, which was assessed by their F1 score in support vector machine (SVM), which was selected as the base model. The result of the highest-ranking feature was determined as the initial one. Each time, a new feature was added, and if the new combination achieved a better result, this new feature was retained, and the initial result was adjusted accordingly. To further analyze the importance of the selected features, SHapley Additive exPlanations (SHAP) (Lundberg and Lee, 2017) was used. SHAP assigns the importance value of each feature by computing its marginal impact across all possible combinations of features in the model predictions.

### 2.4 Automatic score prediction

As presented in Figure 1, this study proposes a task-specific score prediction algorithm, which first explores seven ML models, namely SVM, decision tree (DT), random forest (RF), gradient boosting decision tree (GBDT), logistic regression (LR), Naïve Bayes (NB), and KNN. The model with the highest prediction performance is selected as the optimal single model, and its prediction is named ŷ_*single*_. Afterwards, four ensemble approaches–namely AdaBoost, bagging with soft voting, bagging with hard voting, and stacking–are applied. Except for AdaBoost, which only considers the optimal model, the other three approaches ensemble the optimal model with the other models whose F1 scores differ by less than 0.05 from that of the optimal one. The highest result of the ensemble model is named ŷ_*ensemble*_ and is compared with ŷ_*single*_, with the higher set as the final result. If the two results are equal to each other, the single model is selected as the optimal algorithm, considering the training complexity. For hyperparameter tuning, the Bayesian method (Bergstra et al., 2013) or grid search is selected based on the dimensionality of the hyperparameter space. The hyperparameter tuning result is listed in Table 3.

**Table 3:** Hyperparameter tuning results of each single model for each task.

Models	Hyperparameters	Task									
		FT-R	FT-L	HM-R	HM-L	PS-R	PS-L	TT-R	TT-L	LA-R	LA-L
SVM	*C*: [0.001, 0.01, 0.1, 1, 10, 50, 100, 200]	1	0.001	10	1	50	0.001	1	1	10	0.1
	*gamma*: [0.001, 0.01, 0.1, 1, 5, 10]	0.1	5	0.01	1	0.1	1	0.1	0.01	0.01	1
DT/RF	*max*_*depth*: [1, 2, 3, 4, 5, 6, 7, 8, 9, 10, 15, 20, 30]	9	1	2	20	2	7	6	7	3	1
	*min*_*segments*_*split* : [2, 3, 4, 5, 6, 7, 8, 9, 10]	9	10	8	4	5	2	10	10	2	8
	*min*_*segments*_*leaf* : [2, 3, 4, 5, 6, 7, 8, 9, 10]	4	3	4	4	9	8	4	6	2	4
	*max*_*features*: [1, 2, .., number of features]	2	1	2	2	2	2	6	2	7	5
	*n*_*estimators*: [1, 2, 3, 4, 5, 6, 7, 8, 9, 10, 15, 20, 30]	9	10	11	9	2	10	3	12	7	12
GBDT	*max*_*depth*: [1, 2, 3, 4, 5, 6, 7, 8, 9, 10, 15, 20, 30]	2	1	1	7	1	4	1	3	9	15
	*min*_*segments*_*split*: [2, 3, 4, 5, 6, 7, 8, 9, 10]	4	3	5	9	10	9	3	8	7	2
	*min*_*segments*_*leaf* : [2, 3, 4, 5, 6, 7, 8, 9, 10]	2	8	8	7	10	5	6	10	6	5
	*max*_*features*: [1, 2, .., number of features]	7	1	3	2	2	1	6	2	3	1
	*n*_*estimators*: [1, 2, 3, 4, 5, 6, 7, 8, 10, 15, 20, 30]	6	6	10	12	1	10	11	6	7	10
	*learning*_*rate*: [0.001, 0.005, 0.01, 0.05, 0.1, 0.5]	0.05	0.005	0.05	0.5	0.005	0.1	0.5	0.001	0.01	0.5
LR	*penalty*: [l2, none]	none	l2	l2	none	l2	none	none	l2	l2	l2
	*solver*: [lbfgs, newton-cg, sag, saga]	lbfgs	sag	sag	saga	sag	lbfgs	sag	lbfgs	sag	lbfgs
	*C*: [0.001, 0.005, 0.01, 0.05, 0.1, 0.5, 1, 2, 5, 10, 100]	100	0.05	0.005	0.5	0.001	0.01	0.5	0.005	0.01	0.001
KNN	*n*_*neighbors*: [1, 2, 3, 4, 5]	4	5	2	1	3	1	1	3	4	1
NB	*var*_*smoothing*: [1, 0.5, 0.2, 0.1, 0.05, 0.02, 0.01, 0.005, 0.002, 0.001, 0.0005, 0.0002, 0.0001]	0.0001	1	0.2	0.02	0.02	0.001	0.01	0.01	0.5	1

The McNemar test was applied to statistically analyze whether the optimal algorithm demonstrates significantly superior performance compared to the other models or the ensembling method. The *p*-value was set to 0.05, with Bonferroni correction included for multiple comparisons.

The algorithms were trained and tested in leave-one-subject-out (LOSO) mode. The dataset was divided into 29 folds, each containing the data of a single participant. In each iteration, one fold was used for testing, and the remaining 28 folds were combined for training. To address data imbalance, training data was oversampled using the synthetic minority oversampling technique (SMOTE) (Chawla et al., 2002), with each class accounting for 25%.

This study focuses on score prediction in three scenarios:

i. Precise score prediction. The scores were classified into four scores (0, 1, 2, and 3) according to the UPDRS guidelines. This prediction method was valuable for managing treatment and healthcare plans.
ii. Abnormal status prediction. The scores were categorized into binary classes: normal vs. abnormal [0 vs. (1, 2, 3)] to detect the onset of bradykinesia, which was applied to the preliminary screening for PD.
iii. Critical status prediction. The scores were classified into two levels: normal-mild vs. critical [(0, 1, 2) vs. 3], focusing more on tracking critical status that severely impacted the quality of daily living.

The predicted score for a task was determined by the majority of the results of its segments. The results were evaluated at the event level in two aspects: prediction performance and prediction error. The criteria for assessing prediction involve accuracy, precision, recall, and the weighted average F1 score. The F1 score was chosen as the primary evaluation metric for the imbalanced dataset. The prediction error was evaluated using the root mean square error (RMSE) and the area under the curve (AUC). The 95% confidence interval for each criterion was estimated by stratified Bootstrap by category, resampling 2,000 times. All data analysis processes were performed on the Python 3 platform (Python Software Foundation, Wilmington, USA).

## 3 Results

### 3.1 Score prediction

**Figure 3:**
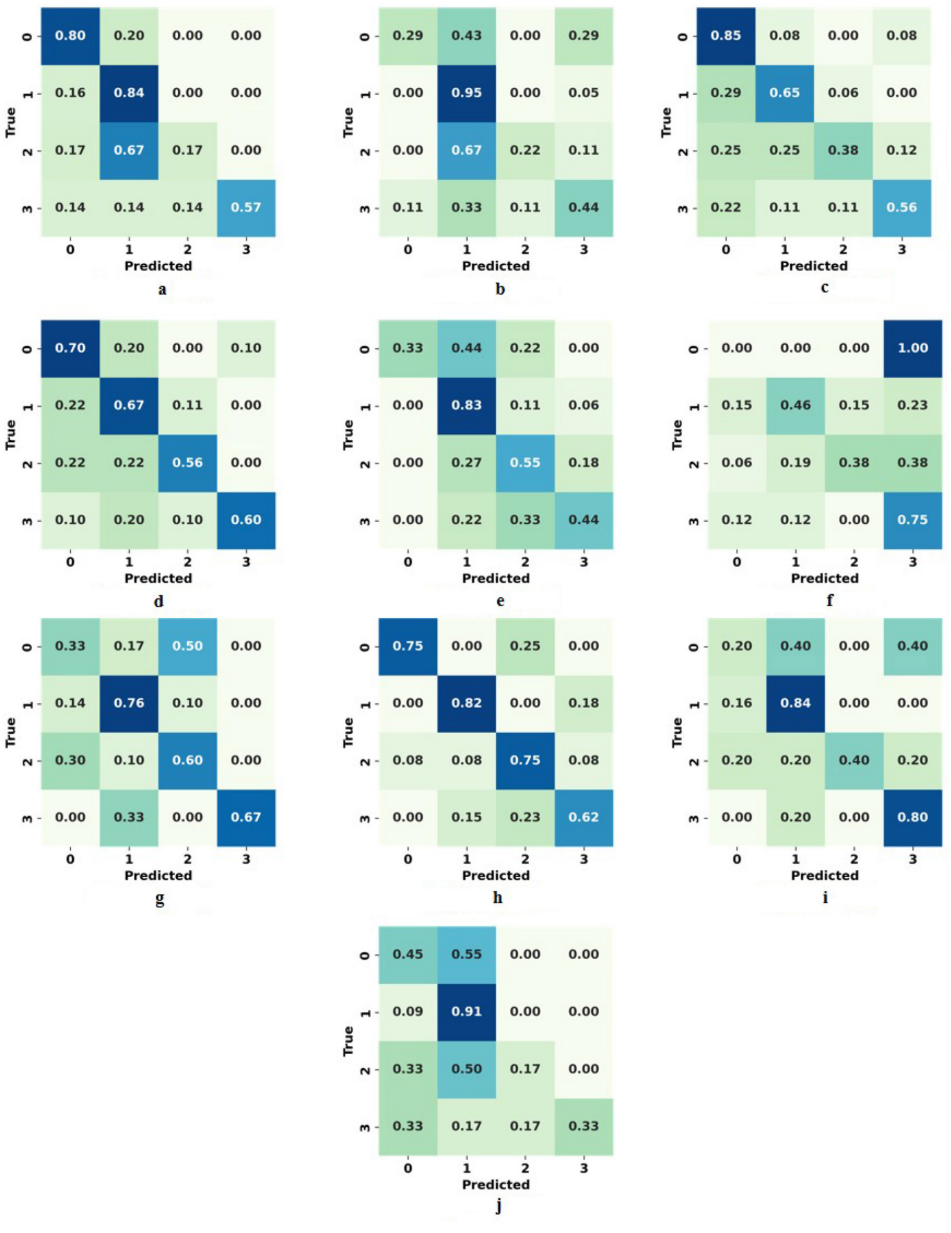
Confusion matrix of the optimal algorithm in precision score prediction (0 vs. 1 vs. 2 vs. 3) for the records of each task. **(a)** FT-R. **(b)** FT-L. **(c)** HM-R. **(d)** HM-L. **(e)** PS-R. **(f)** PS-L. **(g)** TT-R. **(h)** TT-L. **(i)** LA-R. **(j)** LA-L.

**Figure 4:**
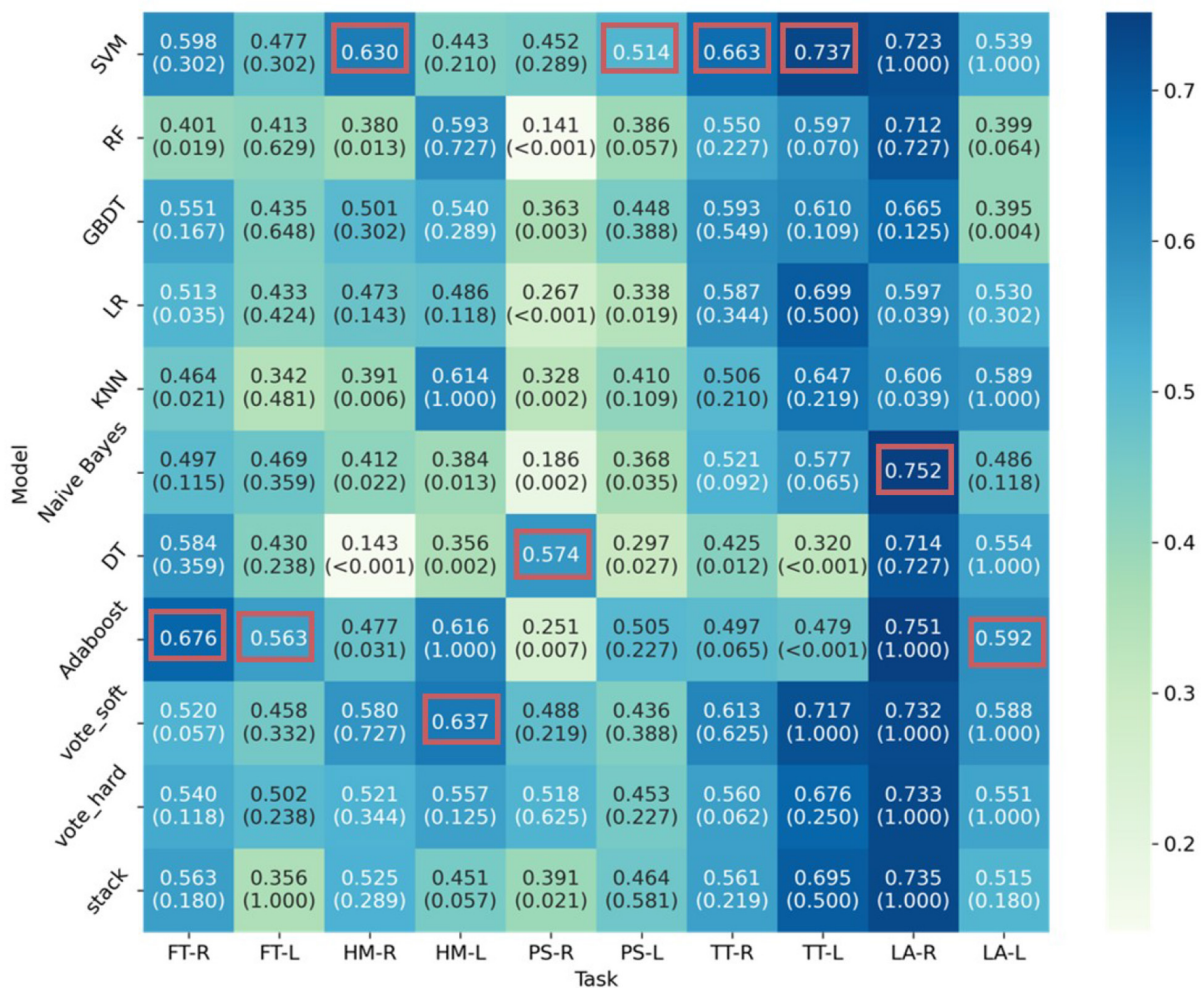
F1 score of all algorithms for each task in precise score prediction. The *p*-value of the MCNemar test between the optimal algorithm and the other models is shown, followed by each F1 score. The highest F1 score is highlighted with a red box.

The results of the first scenario (precise score prediction) of the optimal model for each task are presented in Table 4 and Figure 3. Among the 10 tasks, LA-R achieved the highest performance, with an F1 score of 0.7518 (0.6247–0.8678), an RMSE of 0.8295, and an AUC of 0.8432. The lowest prediction result was for PS-L, with an F1 score of 0.5142 (0.3705–0.6467), an RMSE of 1.2406, and an AUC of 0.5797. Figure 4 presents the F1-score of all algorithms for each task. Among the algorithms, SVM achieved the highest F1-score in four tasks, followed by Adaboost in three tasks, then soft voting, DT, and NB. These optimal models for every task were marginally higher than the other models. For example, although Adaboost achieved the highest F1 score in FT-R, its performance was not significantly better than SVM.

**Table 4:** Score prediction results (average value and confidence interval) of the optimal algorithm in precision score prediction (0 vs. 1 vs. 2 vs. 3) for each task.

Task	Model	Accuracy	Precision	Recall	F1 score	RMSE	AUC
FT-R	Adaboost (DT)	0.7025	0.7034	0.7013	0.6759	0.7580	0.7525
		(0.5952–0.8085)	(0.5699–0.8270)	(0.5957–0.8085)	(0.5498–0.7999)	(0.5053–1.0106)	(0.6510–0.8547)
FT-L	Adaboost (NB)	0.6171	0.6219	0.6163	0.5631	1.0706	0.5822
		(0.5106–0.7234)	(0.4165–0.7783)	(0.5106–0.7234)	(0.4349–0.7000)	(0.7855–1.3369)	(0.4364–0.7259)
HM-R	SVM	0.6386	0.6646	0.6380	0.6299	1.0135	0.7395
		(0.5106–0.7660)	(0.5092–0.7987)	(0.5106–0.7660)	(0.4841–0.7651)	(0.6842–1.3128)	(0.6271–0.8460)
HM-L	Soft voting (GBDT_KNN)	0.6410	0.6719	0.6390	0.6366	0.9698	0.7734
		(0.5106–0.7872)	(0.5336–0.7919)	(0.5106–0.7660)	(0.5040–0.7660)	(0.6684–1.2632)	(0.6575–0.8798)
PS-R	DT	0.5965	0.6506	0.5957	0.5744	0.8481	0.6553
		(0.4681–0.7234)	(0.4950–0.7679)	(0.4681–0.7234)	(0.4425–0.7138)	(0.6523–1.0213)	(0.5428–0.7639)
PS-L	SVM	0.5109	0.5862	0.5084	0.5142	1.2406	0.5797
		(0.3830–0.6383)	(0.4273–0.7273)	(0.3830–0.6383)	(0.3705–0.6467)	(1.0106–1.4732)	(0.5078–0.6553)
TT-R	SVM	0.6522	0.6980	0.6507	0.6633	0.9606	0.7949
		(0.5217–0.7826)	(0.5919–0.805)	(0.5217–0.7826)	(0.5364–0.7853)	(0.7514–1.1610)	(0.6955–0.8881)
TT-L	SVM	0.7405	0.7492	0.7400	0.7365	0.8382	0.7960
		(0.6087–0.8696)	(0.6176–0.8692)	(0.6087–0.8696)	(0.6024–0.8525)	(0.5893–1.0734)	(0.6579–0.9201)
LA-R	NB	0.7624	0.7834	0.7615	0.7518	0.8295	0.8432
		(0.6522–0.8696)	(0.6319–0.8911)	(0.6304–0.8696)	(0.6247–0.8678)	(0.4890–1.1516)	(0.7434–0.9275)
LA-L	Adaboost (KNN)	0.6310	0.6249	0.6292	0.5917	0.9430	0.6835
		(0.5217–0.7391)	(0.4384–0.7690)	(0.5217–0.7391)	(0.4646–0.719)	(0.6916–1.1703)	(0.5625–0.7955)
Average		0.6493	0.6754	0.6480	0.6337	0.9472	0.7200

The results of scenarios ii. and iii. are listed separately in Tables 5, 6. Compared to precise score prediction, the average F1 score of abnormal status prediction increased from 0.6335 to 0.8425, and RMSE decreased from 0.9479 to 0.3803. The performance in critical status predictions was the highest, with an average F1 score of 0.8722, RMSE of 0.3214, and AUC of 0.8021. Furthermore, with the alteration of the scenario, the optimal approaches also varied; for instance, Adaboost (KNN), DT, and RF were the optimal approaches for LA-L under three conditions.

**Table 5:** Score prediction results (average value and confidence interval) of the optimal algorithm in abnormal status prediction [0 vs. (1, 2, 3)] for each task.

Task	Model	Accuracy	Precision	Recall	F1 score	RMSE	AUC
FT-R	Adaboost (DT)	0.7641	0.8158	0.7644	0.7737	0.4800	0.8840
		(0.6383–0.8723)	(0.7101–0.8985)	(0.6383–0.8723)	(0.6503–0.8756)	(0.3573–0.6014)	(0.7583–0.9833)
FT-L	Adaboost (NB)	0.8716	0.8534	0.8735	0.8462	0.3554	0.4199
		(0.8085–0.9362)	(0.7216–0.9406)	(0.8085–0.9362)	(0.7610–0.9286)	(0.2526–0.4376)	(0.1249–0.7393)
HM-R	SVM	0.7661	0.8248	0.7666	0.7742	0.4800	0.8681
		(0.6383–0.8723)	(0.7307–0.9075)	(0.6383–0.8723)	(0.6572–0.8781)	(0.3573–0.6014)	(0.733–0.9683)
HM-L	SVM	0.8947	0.8937	0.8950	0.8917	0.3203	0.7053
		(0.8085–0.9787)	(0.8019–0.9793)	(0.8085–0.9787)	(0.8047–0.9588)	(0.1459–0.4376)	(0.4081–0.9784)
PS-R	DT	0.8730	0.8837	0.8709	0.8408	0.3561	0.5473
		(0.8085–0.9362)	(0.6537–0.9408)	(0.8085–0.9362)	(0.7229–0.9310)	(0.2526–0.4376)	(0.2909–0.7954)
PS-L	SVM	0.8506	0.9117	0.8516	0.8785	0.3799	1.0000
		(0.7447–0.9362)	(0.9057–0.9158)	(0.7660–0.9362)	(0.8173–0.9259)	(0.2526–0.4838)	(1.0000–1.0000)
TT-R	GBDT	0.8064	0.8186	0.8034	0.8101	0.4396	0.6269
		(0.6957–0.8913)	(0.7391–0.9053)	(0.6957–0.8913)	(0.7257–0.9002)	(0.3297–0.5517)	(0.3000–0.9375)
TT-L	SVM	0.9563	0.9588	0.9554	0.9559	0.1929	0.7759
		(0.8913–1.0000)	(0.8943–1.0000)	(0.8913–1.0000)	(0.8967–1.0000)	(0.0000–0.3297)	(0.3512–1.0000)
LA-R	NB	0.8259	0.8311	0.8265	0.8259	0.4134	0.8086
		(0.7174–0.9348)	(0.7155–0.9349)	(0.7174–0.9348)	(0.7107–0.9343)	(0.2554–0.5316)	(0.6653–0.9290)
LA-L	DT	0.8483	0.8474	0.8471	0.8281	0.3857	0.5323
		(0.7609–0.9348)	(0.7283–0.9399)	(0.7609–0.9348)	(0.7159–0.9310)	(0.2554–0.4890)	(0.3182–0.7403)
Average		0.8457	0.8639	0.8454	0.8425	0.3803	0.7168

**Table 6:** Score prediction results (average value and confidence interval) of the optimal algorithm in critical status prediction [(0, 1, 2) vs. 3] for each task.

Task	Model	Accuracy	Precision	Recall	F1 score	RMSE	AUC
FT-R	Adaboost (DT)	0.8304	0.7830	0.8303	0.7963	0.4102	0.8381
		(0.766–0.8936)	(0.7125–0.9054)	(0.7660–0.8936)	(0.7383–0.8672)	(0.3262–0.4838)	(0.6714–0.9643)
FT-L	NB	0.8514	0.8556	0.8515	0.8058	0.3846	0.5072
		(0.8085–0.9149)	(0.6537–0.9230)	(0.8085–0.9149)	(0.7229–0.9049)	(0.2917–0.4376)	(0.2807–0.7339)
HM-R	Soft voting (SVM_GBDT)	0.8935	0.8934	0.8936	0.8916	0.3175	0.7723
		(0.8085–0.9574)	(0.8006–0.9793)	(0.8085–0.9574)	(0.8041–0.9590)	(0.2063–0.4376)	(0.5438–0.9678)
HM-L	GBDT	0.8948	0.8944	0.8949	0.8837	0.3195	0.7946
		(0.8085–0.9574)	(0.7907–0.9596)	(0.8085–0.9574)	(0.7827–0.9574)	(0.2063–0.4376)	(0.5729–0.9703)
PS-R	SVM	0.8495	0.8411	0.8501	0.8356	0.3820	0.6941
		(0.7660–0.9362)	(0.7253–0.9408)	(0.7660–0.9362)	(0.7373–0.9310)	(0.2526–0.4838)	(0.4766–0.8830)
PS-L	GBDT	0.8511	0.8525	0.8497	0.8510	0.3806	0.8352
		(0.7447–0.9362)	(0.7447–0.9418)	(0.7447–0.9362)	(0.7447–0.9373)	(0.2526–0.5053)	(0.6895–0.9556)
TT-R	Soft voting (SVM_GBDT)	0.9350	0.9355	0.9342	0.9323	0.2444	0.9438
		(0.8696–1.0000)	(0.8616–1.0000)	(0.8696–1.0000)	(0.8543–1.0000)	(0.0000–0.3901)	(0.8619–0.9970)
TT-L	NB	0.8259	0.8394	0.8272	0.8292	0.4109	0.8345
		(0.7174–0.9130)	(0.7281–0.9342)	(0.7174–0.9348)	(0.7090–0.9340)	(0.2554–0.5316)	(0.6597–0.9674)
LA-R	DT	0.9778	0.9791	0.9778	0.9754	0.1155	1.0000
		(0.9348–1.0000)	(0.9392–1.0000)	(0.9348–1.0000)	(0.9220–1.0000)	(0.0000–0.2554)	(1.0000–1.0000)
LA-L	RF	0.9342	0.9379	0.9342	0.9210	0.2492	0.8016
		(0.8913–0.9783)	(0.9034–0.9788)	(0.8913–0.9783)	(0.8557–0.9774)	(0.1474–0.3297)	(0.4708–0.9917)
Average		0.8844	0.8812	0.8844	0.8722	0.3214	0.8021

### 3.2 Optimal sensor placement

To investigate optimal sensor placement, we focused exclusively on two types of tasks: PS and LA. The performance of sensors attached to the fingers and toes was evaluated. As shown in Table 7, wrist sensors achieved an average F1-score approximately 0.2400 higher than finger sensors for the PS tasks, and the differences were statistically significant, demonstrating their superior sensitivity in capturing relevant movements. Similarly, sensors on the ankle outperformed those on the toes in LA tasks, though the difference in F1-score in LA-R did not reach statistical significance.

**Table 7:** Comparison the results (mean and confidence interval) of different sensors in evaluating PS and LA tasks in crical status prediction [(0, 1, 2) vs. 3].

Task	Sensor	Accuracy	Precision	Recall	F1 score	RMSE	AUC	*p*-value^*a*^
PS-R	Finger	0.8495	0.8411	0.8501	0.8356	0.3820	0.6941	0.0005
		(0.7660–0.9362)	(0.7253–0.9408)	(0.7660–0.9362)	(0.7373–0.9310)	(0.2526–0.4838)	(0.4766–0.8830)	
	Wrist	0.5938	0.6108	0.5960	0.6026	0.6341	0.4855	
		(0.4894–0.7021)	(0.5811–0.6353)	(0.4894–0.7021)	(0.5156–0.6670)	(0.5458–0.7293)	(0.2749–0.6901)	
PS-L	Finger	0.8511	0.8525	0.8497	0.8510	0.3806	0.8352	0.0074
		(0.7447–0.9362)	(0.7447–0.9418)	(0.7447–0.9362)	(0.7447–0.9373)	(0.2526–0.5053)	(0.6895–0.9556)	
	Wrist	0.6180	0.5966	0.6184	0.6013	0.6192	0.6407	
		(0.5106–0.7447)	(0.465–0.7358)	(0.4894–0.7447)	(0.4803–0.7212)	(0.5053–0.7146)	(0.4657–0.7904)	
LA-R	Toe	0.9778	0.9791	0.9778	0.9754	0.1155	1.0000	0.0730
		(0.9348–1.0000)	(0.9392–1.0000)	(0.9348–1.0000)	(0.9220–1.0000)	(0.0000–0.2554)	(1.0000–1.0000)	
	Ankle	0.8493	0.8640	0.8485	0.8517	0.3863	0.6984	
		(0.7609–0.9348)	(0.7826–0.9458)	(0.7391–0.9348)	(0.7703–0.9373)	(0.2554–0.4890)	(0.3999–0.9268)	
LA-L	Toe	0.9342	0.9379	0.9342	0.9210	0.2492	0.8016	0.0018
		(0.8913–0.9783)	(0.9034–0.9788)	(0.8913–0.9783)	(0.8557–0.9774)	(0.1474–0.3297)	(0.4708–0.9917)	
	Ankle	0.6740	0.7840	0.6769	0.7160	0.5658	0.6615	
		(0.5435–0.7826)	(0.7115–0.8619)	(0.5435–0.8043)	(0.6163–0.8142)	(0.4423–0.6757)	(0.4916–0.8250)	

^*a*^The *p*-value is the result of the McNemar test between two sensors using F1 scores.

### 3.3 Representative features

**Figure 5:**
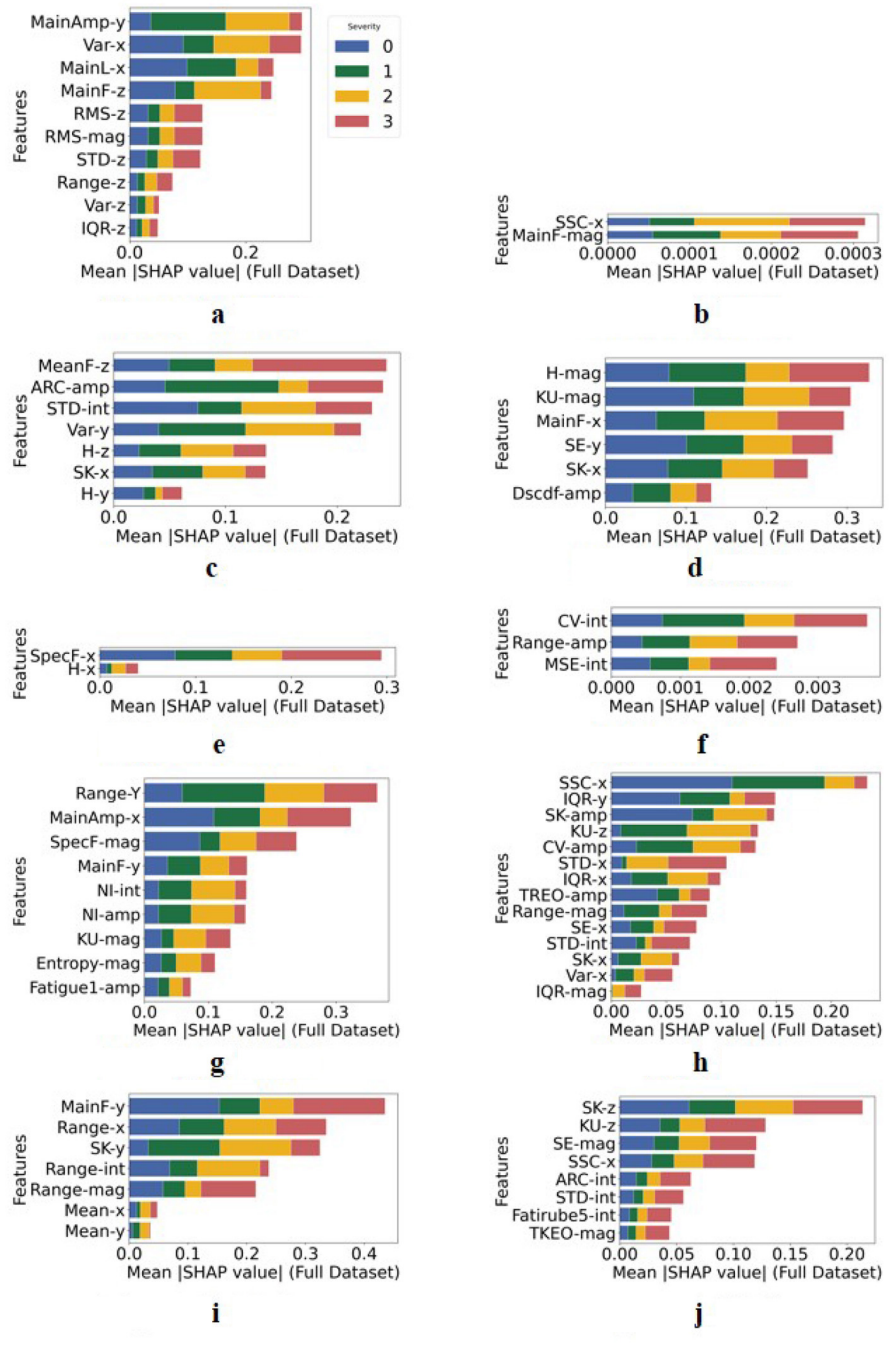
Stacked bar plot of the SHAP values of the selected features for each task in the precise score prediction (0 vs. 1 vs. 2 vs. 3). **(a)** FT-R. **(b)** FT-L. **(c)** HM-R. **(d)** HM-L. **(e)** PS-R. **(f)** PS-L. **(g)** TT-R. **(h)** TT-L. **(i)** LA-R. **(j)** LA-L.

Figure 5 presents the SHAP values of the selected features in precise score prediction for each task. The types of features and their contribution varied among severity levels, tasks, and body sides. In tasks FT-L, PS-R, and PS-L, only two or three features were contributed to the prediction, which also obtained the lowest F1 scores as shown in Figure 4. The task TT-L had the largest number of feature types.

## 4 Discussion

### 4.1 Score prediction

This study evaluated three scoring methods; critical status prediction achieved the best performance, indicating that the proposed system was more capable of differentiating critical status from normal-wild status. The inferior performance of the precise score prediction classification compared to the binary ones could be attributed to the smaller number of segments per class, with a consequent increase in sensitivity to inter-participant variability. Although critical status prediction obtained a relatively high performance, the samples of score 4 were categorized as score 3; thus, the feasibility of the proposed system in identifying the most severe status needs further validation, with a larger dataset.

According to Figure 3, in precise score prediction, score 1 generally obtained the highest performance, except for task HM-R. Most misclassifications occurred between adjacent lower scores. For example, in task PS-R, a considerable portion of scores 0 and 2 were misclassified as score 1. One potential explanation for this was the high similarity in movement characteristics across adjacent statuses, in particular in lower score ranges. Another reason could be the uncertainty in the ratings driven by the inherent ambiguity of clinician scoring.

**Figure 6:**
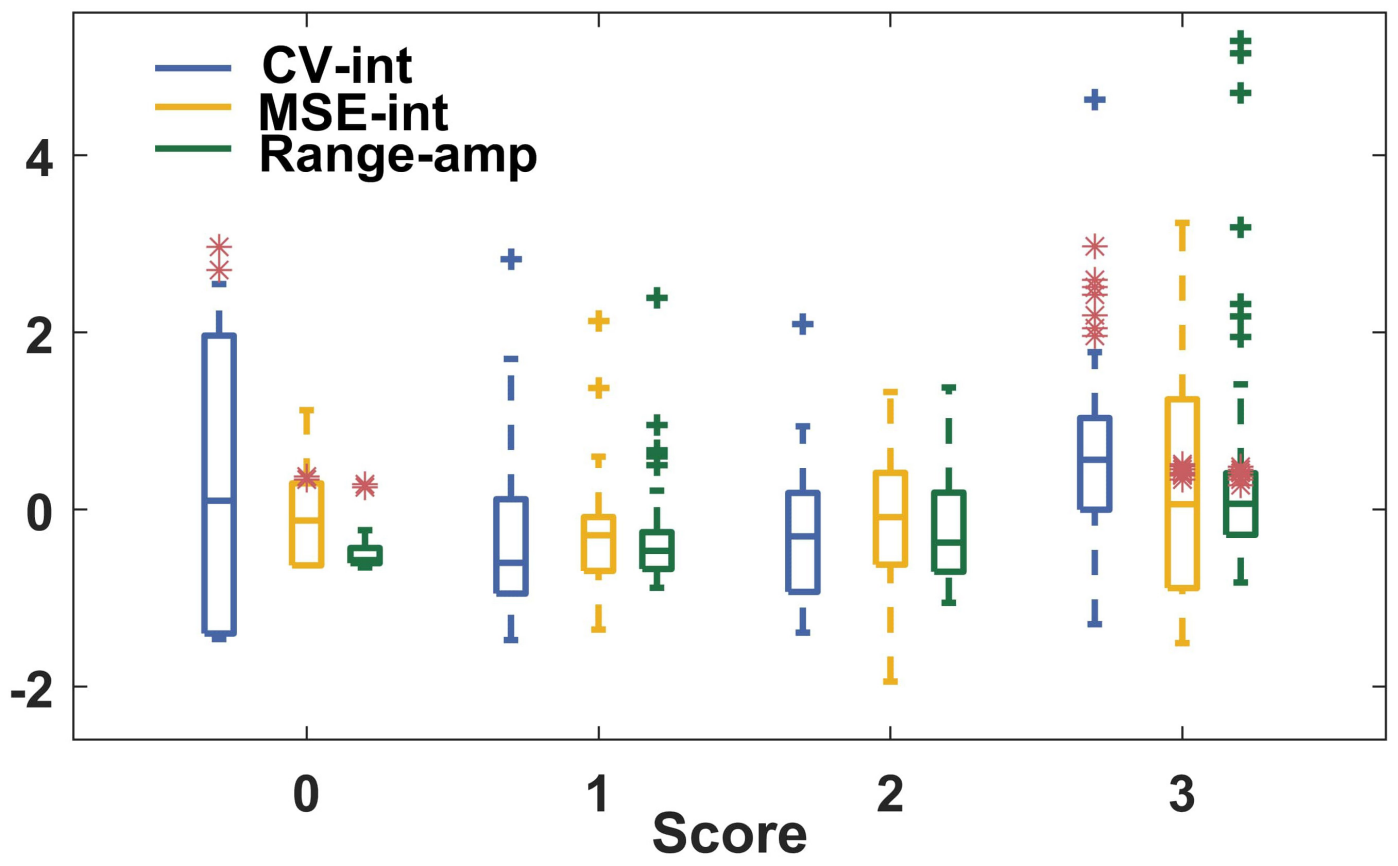
Boxplot of the distribution of the selected features for task PS-L. Features of segments misclassified as scores 0/1 are highlighted with a red asterisk.

Among the tasks, task PS yielded the lowest results in precise score prediction. This suggested a limitation of the ACM in tracking rotational movements. Specifically, scores 0 and 3 were misclassified with each other in task PS-L. As illustrated in Figure 6, the features of the misclassified segments with scores 0 and 1 are in the same range, making the two scores difficult to separate. Furthermore, misclassified score 0 segments came from one female participant aged 55, whereas misclassified score 3 segments were all from male participants with an average age of 69. This may indicate a similarity in functional performance in genders and ages, as suggested by the study of (Keezer et al. 2016), and requires validation in a larger group in a future study.

In contrast to other studies that focus on predicting the UPDRS III score, the proposed system remained efficient. For example, for task FT, its accuracy was at least 0.11 higher than that of study (Islam et al., 2023) in predicting the four scores and 0.04 higher than that of study (Crowe et al., 2024) in predicting normal versus abnormal. Nonetheless, it was limited to predicting the PS score compared to the survey by (Sánchez-Fernández et al. 2023), which could be due to the absence of a GYR and magnetometer.

### 4.2 Representative features

Kinematic features were initially proposed to predict the score of FT (Arora et al., 2015; Stamatakis et al., 2013), and this study verified their efficiency in other tasks. For example, STD-int was selected in predicting the scores for tasks HM-R, TT-L, and LA-L. This could be because of the similar movement patterns of these tasks. In addition, more time-domain and frequency features, for example, MainAmp, SSC, SpecF, and MeanF. were selected, and they contributed more than kinematic features, as shown in Figure 5. Typically, SpecF-x, H-x, CV-int, Range-amp, and MSE-int were verified to be correlated with PS, which were not proposed in the previous study (Sánchez-Fernández et al., 2023). Since these features present variations in velocity or amplitude, it implies that movement stability is a critical parameter in predicting bradykinesia. Although gender and age were not selected, they could be relevant to specific representative characteristics, as proposed by studies (Gamborg et al., 2023; Picillo et al., 2017) that indicated the lower muscle strength of the female and older groups in maintaining movement stability.

Regarding the same task, the representative features vary on both sides, suggesting that the criteria for evaluating tasks on the different sides should be adjusted accordingly. Taking task LA as an example, while scoring the task conducted with the right leg, the general movement conditions, for example, speed, need to be emphasized. In comparison, when evaluating the performance of the left leg, factors such as movement progress, including speed, stability, and fatigue, are more critical. The difference could be attributed to the impact of the dominant side, which warrants further exploration.

### 4.3 Comparison of models

This study designs task-specific algorithms through the exploration of single ML models and ensemble methods. In comparison, ensemble methods did not achieve the highest performance in all the tasks, only in tasks FT-R, FT-L, HM-L, LA-R, and LA-L for precision score prediction; tasks FT for abnormal status prediction; and tasks FT-R, HM-L, and TT-R for critical status prediction. In addition, their performances were not significantly higher than the optimal single models, as shown in Figure 4. This condition was also addressed in previous studies (Džeroski and Ženko, 2004; Hornyák and Iantovics, 2023). One reason could be that, given the small size of the dataset compared to the high individualized differences, with a more complex architecture compared to single models, ensemble methods were more likely to result in overfitting. This could also be the reason for the varied optimal model among tasks and even between the tasks performed by different body sides. Another reason could be the mechanism of the ensemble methods, typically hard-voting and stacking, as their performance depended on that of the integrated single models.

The proposed system was compared with the state-of-the-art studies, as listed in Table 8. To the best of the authors' knowledge, only one study (Singh et al., 2023) has analyzed the same dataset to predict task scores, and its results are listed in Table 8. This study developed a deep neural network, XceptionTime, to predict critic status. To ensure a fair comparison with the study by (Singh et al. 2023), the proposed system was retrained and tested in the dataset, excluding samples scored as 2. Additionally, the accuracies for each task on both sides were averaged. As a result, the proposed system achieved an accuracy of more than 0.15 higher than that of the study (Singh et al., 2023) on all tasks, indicating the effectiveness of the proposed system with this dataset. Furthermore, the physical meaning of the hand-crafted features was more readily interpretable to clinicians.

**Table 8:** Comparison of the accuracies between the proposed system and state-of-the-art studies.

Study	Study by (Singh et al. 2023)	Study by (Sigcha et al. 2022)	Proposed	
Scenario i./iii.	Critic (without score 2)	Precise	Critic (without score 2)	Precise
Dataset	Same as proposed	6 PD, 8 control	21 PD, 8 control	
Sensor type	Same as proposed	ACM + GYR	ACM	
Sensor placement	Same as proposed	Wrist	Index finger, wrist, big toe, and ankle	
Model	Xception-Time	CNN+RF	Proposed system	
Validation method	LOSO	Five-fold cross-validation	LOSO	Five-fold cross-validation
FT	0.680	0.860	0.810	0.721
HM	0.789		0.883	0.821
PS	0.701		0.866	0.670
TT	0.728	–	0.870	0.855
LA	0.773	–	0.950	0.834

The proposed system was compared with the study (Sigcha et al., 2022), which also focused on predicting upper-limb bradykinesia status with ACMs worn on the wrists. Different from the proposed system, this study also incorporated GYRs, and the CNN was employed to extract features for RF. The study by (Sigcha et al. 2022) outperformed the proposed system, particularly in task PS. This reflected the better suitability of GYRs for capturing rotational movements compared to ACMs, which was consistent with the research of (Sánchez-Fernández et al. 2023). However, the model was validated using five-fold cross-validation, which could result in a biased outcome. In addition, the effectiveness of the sensor on the wrist to evaluate finger-related tasks requires further exploration.

### 4.4 Sensor placement position

**Figure 7:**
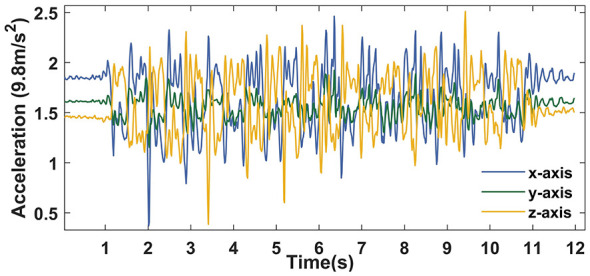
Acceleration signals of task PS-R collected by the sensor on the finger for participant 1.

The results in Table 7 suggest that the sensors attached to the wrists and ankles were significantly less effective than those on the fingers and toes for tasks PS and LA, respectively, further supporting the need to attach sensors to body parts closely related to movement. To further explore the reason, as an example, the raw signals of task PS-R collected by the ACM on the index finger are shown in Figure 7. In contrast, the signals collected on the wrist (Figure 2e) have a higher signal-to-noise ratio.

### 4.5 Future work

The feasibility of the proposed system to automatically predict the bradykinesia severity of PD has been validated. Future efforts will focus on enhancing its performance and usability in three aspects: dataset, sensor, and application.

- Firstly, future work will not only increase the size of the dataset, especially the number of samples for score 4, but also enhance patient diversity, for example, age range and racial background. In addition, multiple clinicians will be involved in scoring to investigate inter-rater variability.
- Secondly, to compensate for the limitations of ACMs in tracking rotational movements, other sensor types will be incorporated, for example, GYRs. As ACMs were necessary to be worn on tracked body parts (Section 4.4), the sensors can be integrated into rings, smartwatches, or smart clothes to minimize the manipulation complexity.
- Finally, building on our previous study (Zhang et al., 2025), the proposed system offers the potential to automatically end-to-end monitor (identify and score) the five UPDRS tasks. With the advancement of sensor technology and the development of guidance software to assist patients and their caregivers, the system's applicability will expand from clinical environments to home settings, enabling continuous bradykinesia assessment.

## 5 Conclusion

This study proposes a task-specific system to automatically predict extremity bradykinesia in PD patients. This system integrates optimal ACM placement positions and automatic score prediction algorithms for each task, even for the same task performed by different sides of the extremity. Ultimately, this system has been verified to be capable of predicting scores for five UPDRS III tasks correlated with bradykinesia. This study implies the potential of automatic prediction of bradykinesia status, which could be valuable for monitoring bradykinesia both in clinical environments and home settings.

## Data Availability

The original contributions presented in the study are included in the article/supplementary material, further inquiries can be directed to the corresponding author.
